# Machine learning-based prediction of one-year mortality after alloHCT identifies the impact of pre-transplant immunity and inflammation

**DOI:** 10.3389/fimmu.2025.1745873

**Published:** 2026-01-19

**Authors:** Thomas Meyer, Robert Meyer, Maren Hackenberg, Daniela Oelke, Laura Gengenbach, Christoph Rummelt, Hauke Wilcken, Kristina Maas-Bauer, Ralph Wäsch, Justus Duyster, Hartmut Bertz, Jesús Duque-Afonso, Jürgen Finke, Robert Zeiser, Claudia Wehr

**Affiliations:** 1 Department of Internal Medicine I, Hematology, Oncology and Stem Cell Transplantation, Medical Center - University of Freiburg, Faculty of Medicine, Freiburg, Germany; 2 Collaborative Research Institute Intelligent Oncology (CRIION), Freiburg, Germany; 3 Alcemy GmbH, Berlin, Germany; 4 Institute of Medical Biometry and Statistics, Faculty of Medicine and Medical Center, University of Freiburg, Freiburg, Germany; 5 Offenburg University of Applied Sciences, Offenburg, Germany; 6 IMMediate Advanced Clinician Scientist-Program, Department of Medicine II, Medical Center – University of Freiburg and Faculty of Medicine, University of Freiburg, Freiburg, Germany

**Keywords:** alloHCT, explainable AI, immunocompetence, inflammation, lymphocytes, machine learning, one-year mortality, risk prediction

## Abstract

Accurate prediction of mortality after allogeneic hematopoietic stem cell transplantation (alloHCT) is essential for individualized treatment decisions, yet existing clinical risk scores capture only a limited number of variables and show modest predictive performance. In our single-center retrospective analysis, we included data from 909 adult patients with hematologic malignancies undergoing alloHCT. We used 31 features to build machine-learning models to predict death within the first year after alloHCT. These features included established clinical risk factors together with pre-transplant lymphocyte subsets and inflammatory markers. Among four models, a random forest algorithm showed the best performance (AUC = 0.773) and retained good generalizability in an independent test set (AUC = 0.748). SHapley Additive exPlanations (SHAP)-based interpretation of the machine-learning models showed that age together with five easily measurable pre-transplant immunological and inflammatory parameters influenced the outcome: pre-transplant CD4^+^, CD8^+^, and B-lymphocyte counts, albumin, and C-reactive protein (CRP) levels. Based on these features, our random forest approach outperformed established clinical risk scores (HCT-CI, EASIX, rDRI, mGPS) in predicting one-year mortality after alloHCT and more effectively distinguished patients at low and high risk of an adverse outcome. Our study shows that machine-learning-based models can not only predict patient outcomes after alloHCT but also serve as powerful tools for data exploration, confirming the prognostic relevance of pre-transplant inflammation while uncovering the critical role of lymphocyte subsets as previously unknown risk factors. External validation in independent multicenter cohorts will be required to confirm generalizability.

## Introduction

Allogeneic hematopoietic stem cell transplantation (alloHCT) is a potentially curative treatment option for patients with hematologic malignancies. Over recent decades, survival following alloHCT has steadily improved, with current estimates showing ~20-30% overall mortality and ~15% non-relapse mortality within the first year post-transplant (1). Accurately estimating individual mortality risk after alloHCT is essential for informed decision-making by both physicians and patients, and also plays a critical role in safeguarding donors. Several prognostic scores have been developed to predict overall and/or non-relapse mortality (2–7). The Hematopoietic Cell Transplantation Comorbidity Index (HCT-CI) quantifies comorbidity burden and predicts non-relapse mortality, and is also widely used to estimate overall survival (7, 8). The refined Disease Risk Index (rDRI) reflects disease-related risks (2). The Endothelial Activation and Stress Index (EASIX) assesses endothelial dysfunction (5, 6), and the modified Glasgow Prognostic Score (mGPS) uses albumin and CRP for outcome prediction (9, 10). However, current scoring systems lack the desirable accuracy for individualized outcome prediction after alloHCT, as demonstrated by reported AUC values for one-year survival prediction typically ranging between 0.53 and 0.64 (8).

To address the potential limitations in the complexity of traditional scores, machine learning-based approaches have been implemented for mortality prediction, demonstrating promising results (11–15). Nevertheless, these models lack the explainability aspect of a scoring system. Machine learning has also been employed to explore the impact of individual features on relapse or mortality risk (16–18). While the prognostic relevance of pre-transplant immunological factors is increasingly recognized (9), most studies have focused on post-transplant immune reconstitution and its association with transplantation characteristics. In parallel, accumulating evidence highlights that a heightened systemic inflammatory state before transplantation represents an important and independent determinant of post-transplant outcomes (19–23).

In this single-center study of 909 adults undergoing alloHCT, we set out to identify new, accessible predictors, focusing on immunological features and incorporating emerging inflammatory risk factors, and to determine their relative importance for one-year mortality after transplantation using SHapley Additive exPlanations (SHAP) values (24, 25). We applied machine learning to analyze pre-transplant disease, donor, and recipient factors, including lymphocyte subset counts and inflammatory markers to predict one-year mortality within a rigorous nested cross-validation framework and independent test validation step. The approach yielded a well-performing, disease-agnostic generalizable model. Model interpretation underscored the prognostic value of pre-transplant immune and inflammatory status, which we benchmarked against established clinical risk scores (HCT-CI, rDRI, EASIX, mGPS) demonstrating superior predictive performance of our model.

## Methods

### Patient and donor characteristics

This section describes the patient cohort, variable definitions, and data sources used for model development. The work was conducted in accordance with the tenets of the Declaration of Helsinki, approved by the local ethics committee (EK-FR: 22-1490-S1-retro). Informed consent for data analysis was obtained from all patients. We retrospectively identified all patients who underwent first alloHCT between 2008 and 2023 and for whom lymphocyte subsets data were available in our institutional database. Among 1,346 first alloHCT recipients, 909 patients with available lymphocyte subset data were included (**Table 1**, **Supplementary Figure S1A**). Data were extracted retrospectively from our prospectively maintained database. The 31 features used in model development are shown in **Table 2** and represent routinely available variables across transplant centers.

**Table 1:** Patient characteristics.

Variable	Value	Variable	Value
N = 909			
Year of alloHCT	2016 (2008–2023) [Range]	Pre-Transplant risk scores	
Age at alloHCT	58 (18–79) [Range]	HCT-CI	
Donor Age	33 (14–71) [Range]	0	199 (21.9%)
Disease Features		1–2	262 (28.9%)
Myeloid Disease	591 (65.0%)	≥3	447 (49.2%)
Lymphoid Disease	287 (31.6%)	mGPS	
AML	411 (45.2%)	0	637 (70.9%)
MDS	96 (10.6%)	1	182 (20.3%)
ALL	83 (9.1%)	2	79 (8.8%)
Lymphoma	124 (13.6%)	EASIX	1.79 (0.90–5.10) [IQR]
Multiple Myeloma	80 (8.8%)	rDRI	
Disease not in Remission (active)	554 (60.9%)	Low	28 (3.3%)
Conditioning Intensity TCI score	2.50 (1.50–4.50) [Range]	Intermediate	407 (47.4%)
GvHD Prophylaxis		High	329 (38.3%)
ATLG	500 (55.0%)	Very High	94 (11.0%)
PT-CY	73 (8.0%)	Cause of Death	
Ciclosporin A (CyA)	859 (94.5%)	Progressive Disease	224 (24.6%)
Everolimus	52 (5.7%)	Infection	135 (14.9%)
Mycophenolic acid (MPA)	644 (70.8%)	GvHD	28 (3.1%)
Methotrexate	84 (9.2%)	Vascular event	17 (1.9%)
Alemtuzumab	184 (20.2%)	Secondary malignancy	13 (1.4%)
GvHD Grade		other	44 (4.8%)
aGvHD >Grade 1	318 (35.0%)		
cGvHD moderate or worse	273 (30.0%)		
Transplant variables			
Patient Gender male	545 (60.0%)		
Donor Gender male	576 (63.4%)		
Matched Related Donor (MRD)	219 (24.1%)		
Mismatched Related Donor (MMRD)	38 (4.2%)		
Matched Unrelated Donor (MUD)	451 (49.6%)		
Mismatched Unrelated Donor (MMUD)	199 (21.9%)		
Syngen	2 (0.2%)		

Patient, disease, and transplant characteristics of the study cohort (n = 909).

Continuous variables are shown as median (min–max) [Range] or median (25th–75th percentile) [IQR]. Categorical variables are presented as count (percentage).

**Table 2:** Univariate analysis of 31 features used for machine learning models.

Variable	Overall	1-year survivors (n=677)	death within the 1st year (n=232)	p-value
Age at alloHCT	58.5 (48.3–66.1)	57.0 (45.3–64.8)	63.3 (54.1–68.7)	<0.001
Donor Age	33.0 (25.0–47.0)	32.0 (25.0–46.0)	36.0 (27.0–49.0)	0.003
TCI score	2.5 (2.5–3.5)	2.5 (2.5–3.5)	2.5 (2.5–2.5)	0.012
Performance status at alloHCT	90.0 (80.0–90.0)	90.0 (80.0–90.0)	80.0 (80.0–90.0)	<0.001
Disease Features				
AML	411/909 (45.2%)	304/677 (44.9%)	107/232 (46.1%)	0.748
MDS	96/909 (10.6%)	81/677 (12.0%)	15/232 (6.5%)	0.019
Lymphoma	124/909 (13.6%)	68/677 (10.0%)	56/232 (24.1%)	<0.001
Active disease at alloHCT	554/909 (60.9%)	382/677 (56.4%)	172/232 (74.1%)	<0.001
Lymphocytes				
pre-transplant B-cells (cells/µl)	24.0 (2.0–90.0)	32.0 (3.0–99.0)	10.5 (1.0–69.5)	<0.001
pre-transplant CD4+ T cells (cells/µl)	343.0 (209.0–524.0)	375.0 (226.0–541.0)	280.5 (167.5–435.4)	<0.001
pre-transplant NK cells (cells/µl)	89.0 (40.0–158.0)	93.0 (41.0–166.0)	77.0 (38.0–138.2)	0.071
pre-transplant CD8+ T cells (cells/µl)	249.0 (137.0–442.0)	264.0 (151.0–445.0)	201.2 (109.5–387.2)	0.005
Laboratory values				
LDH (U/L)	200.0 (169.0–271.5)	196.5 (169.0–257.0)	222.0 (169.8–321.9)	0.003
Platelets (K/µL)	111.0 (42.0–185.5)	122.0 (49.0–199.0)	76.2 (29.4–159.5)	<0.001
Creatinine (mg/dL)	0.9 (0.7–1.0)	0.8 (0.7–1.0)	0.9 (0.8–1.1)	0.002
Albumin (g/dL)	4.1 (3.8–4.3)	4.1 (3.8–4.4)	3.8 (3.5–4.2)	<0.001
CRP (mg/L)	4.2 (0.0–12.5)	3.5 (0.0–10.0)	8.0 (1.5–20.0)	<0.001
ALT (GPT) (U/L)	25.0 (16.0–42.5)	26.0 (16.0–42.0)	25.0 (15.2–45.0)	0.75
GvHD prophylaxis				
ATLG	500/909 (55.0%)	391/677 (57.8%)	109/232 (47.0%)	0.004
PT-CY	73/909 (8.0%)	49/677 (7.2%)	24/232 (10.3%)	0.133
CyA	859/909 (94.5%)	640/677 (94.5%)	219/232 (94.4%)	0.937
Everolimus	52/909 (5.7%)	37/677 (5.5%)	15/232 (6.5%)	0.571
MPA	644/909 (70.8%)	489/677 (72.2%)	155/232 (66.8%)	0.117
Methotrexate	84/909 (9.2%)	69/677 (10.2%)	15/232 (6.5%)	0.091
Alemtuzumab	184/909 (20.2%)	119/677 (17.6%)	65/232 (28.0%)	<0.001
Patient/donor				
Patient Gender Male	545/909 (60.0%)	396/677 (58.5%)	149/232 (64.2%)	0.124
Donor Gender Male	576/909 (63.4%)	426/677 (62.9%)	150/232 (64.7%)	0.637
Mismatch	237/909 (26.1%)	154/677 (22.7%)	83/232 (35.8%)	<0.001
CMV high risk (R+)	504/909 (55.4%)	372/677 (54.9%)	132/232 (56.9%)	0.606
CMV int. risk (D+/R-)	94/909 (10.3%)	63/677 (9.3%)	31/232 (13.4%)	0.08
CMV low risk (D-/R-)	311/909 (34.2%)	242/677 (35.7%)	69/232 (29.7%)	0.096

Comparison between survivors and non-survivors one year after alloHCT. Continuous variables are reported as median (interquartile range; 25th-75th percentile) and compared using the Mann-Whitney U test. Categorical variables are presented as count/total (percentage) and compared using the chi-square test.

We defined any disease status other than a partial response (PR) or complete response (CR) recorded before alloHCT as active disease. For this study, donor-recipient mismatch was defined as a 5–9 out of 10 human leukocyte antigen (HLA) match. Conditioning intensity was quantified using the Transplant Conditioning Intensity (TCI) score, calculated according to published criteria (26). Lymphocyte subsets were measured as described previously (21) between day -20 and day -6 before the start of conditioning for alloHCT. For laboratory parameters, we included lactate dehydrogenase (LDH), platelet count, creatinine, albumin, C-reactive protein (CRP), and alanine aminotransferase (ALT). For each patient, the median value within the same pre-transplant time window (day -20 to -6) was used.

In addition to these variables, we assessed four established clinical scores: the Hematopoietic Cell Transplantation-Comorbidity Index (HCT-CI) (7), the refined Disease Risk Index (rDRI) (2), the Endothelial Activation and Stress Index (EASIX) (27), and the modified Glasgow Prognostic Score (mGPS) (9, 10). These scores were computed according to their published definitions. For the EASIX and mGPS scores, the above-mentioned median laboratory values were used for calculation. For Kaplan-Meier analyses concerning EASIX, we applied the thresholds published by Shouval et al. (8), which approximately correspond to the quantile-based categories used in other studies (27, 28). One patient had a missing HCT-CI value due to incomplete pulmonary function testing, 11 patients had missing mGPS values owing to unavailable pre-transplant albumin measurements, and 51 patients had missing rDRI scores because the index was not applicable to their underlying disease according to its definition (2).

### Model generation and feature analysis

**Figure 1:**
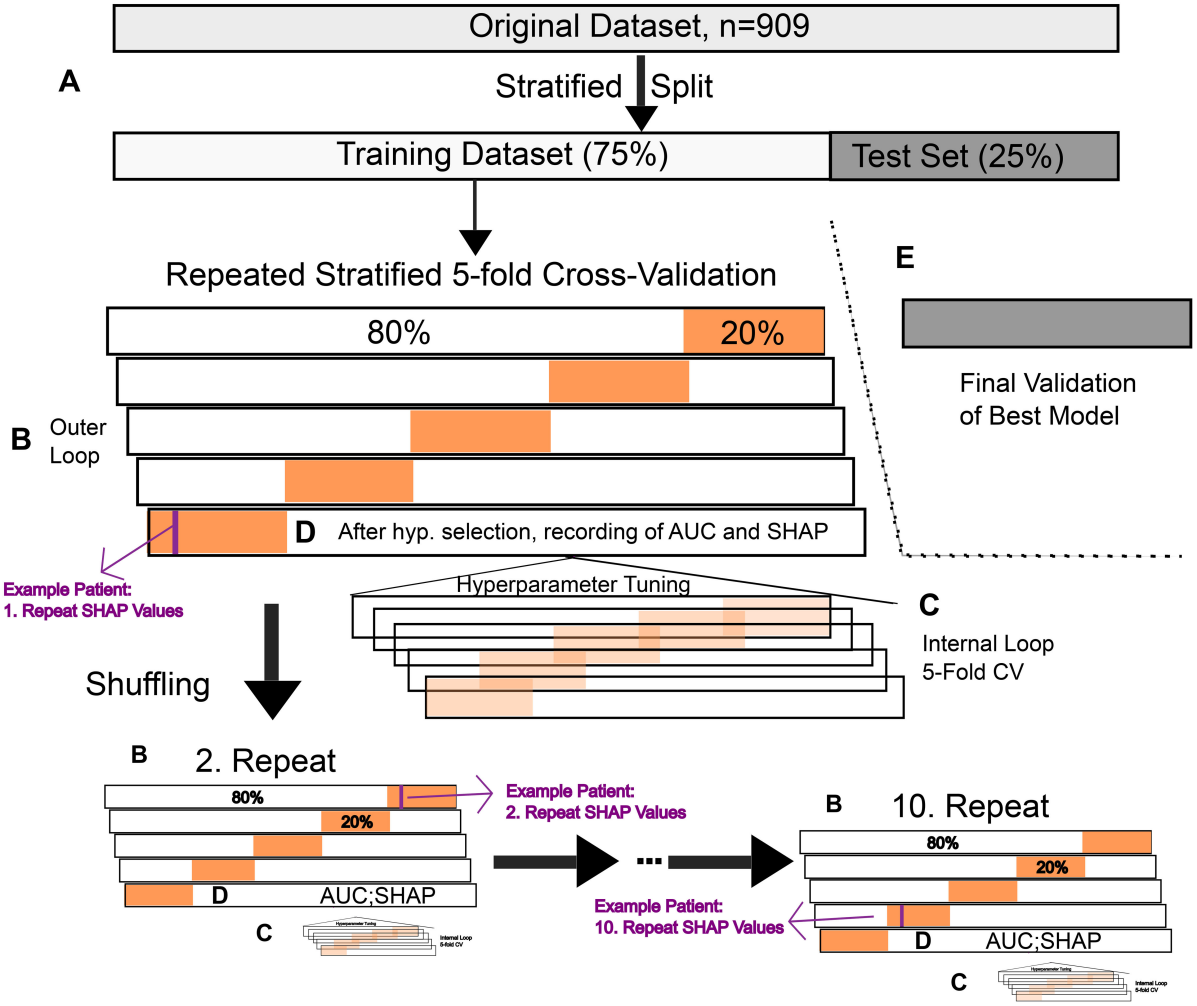
Visualization of model generation. The cohort (n = 909) was split 75/25 into a training set and a locked test set (gray, **A**). The training data underwent 10 × repeated 5-fold nested cross-validation (orange): The outer folds **(B)** supplied 50 AUC estimates per algorithm and patient-level SHAP values **(D)**, while the inner folds were used to tune hyperparameters **(C)**. SHAP values were recalculated whenever a patient re-entered an outer training fold and then averaged (example in purple). The final model was refitted on the complete training set and evaluated once on the independent test set to confirm generalizability **(E)**.

To ensure transparent, unbiased, and reproducible model development, we implemented a machine-learning pipeline combining nested cross-validation, SHAP-based feature selection, and hyperparameter optimization (**Figure 1**). The study was conducted in accordance with the TRIPOD-AI (Transparent Reporting of a multivariable prediction model for Individual Prognosis or Diagnosis-Artificial Intelligence extension) guidelines (29). A version of the TRIPOD-AI checklist is provided in the **Supplementary Materials** (**Supplementary Table S1**).

To develop and evaluate predictive models for one-year survival after alloHCT, we implemented a machine-learning pipeline combining nested cross-validation, SHAP-based feature selection, and hyperparameter optimization (**Figure 1**). We selected death within the first year after alloHCT as the primary categorical endpoint for model development. This time frame was chosen because of its clinical relevance and relatively high event frequency, enabling robust statistical modeling based on baseline variables. Moreover, early mortality is often strongly influenced by pre-transplant patient-, donor-, and disease-related factors, making it a suitable outcome for prediction based on baseline variables. Follow-up was complete during the first year after transplantation, with no censoring before the one-year time point. Categorical features were one-hot encoded. The overall degree of missingness was low, with only 14 missing values out of 28,179 data points. Median imputation was applied where necessary for all models except XGBoost, which inherently handles missing values. Imputation was implemented within the machine-learning pipeline and therefore performed separately within each cross-validation training fold, with imputer parameters learned exclusively from the respective training data and applied to the corresponding validation or test folds, thereby preventing information leakage. Imputation affected four variables: pre-transplant albumin (n=11), pre-transplant ALT (n=1), and donor gender and donor age (n=1 each). The single missing values for donor gender and donor age arose from a cord blood transplantation, for which donor characteristics (sex and age) are not consistently defined or recorded.

We compared four algorithms: logistic regression, gradient boosting, XGBoost, and random forest. Before model generation, a 75/25 stratified data split was performed to maintain an event frequency of ~25% in both the 75% training and the 25% test set (**Figure 1A**). The test set was only used after model selection to ensure generalizability.

Using the training set, we performed 10 repeats of 5-fold nested cross-validation. The outer loop (**Figure 1B**) yielded unbiased performance estimates. For each of the 50 outer folds, we refit the model with the hyperparameters selected in the inner loop, and both the area under the receiver operating characteristic curve (AUC) and patient-level SHAP values were computed (**Figure 1D**). Within each outer training split, hyperparameters were optimized by randomized search over a predefined grid using 5-fold cross-validation with receiver operating characteristic (ROC) AUC scoring (**Figure 1C**).

To avoid information leakage and ensure unbiased feature selection, feature selection was performed within the inner cross-validation loop using a SHAP-based approach. Feature selection was done inside this inner cross-validation via a SHAP-based selector implemented in the pipeline: a lightweight model matching the main algorithm was fit on each inner training fold, features were ranked by mean absolute SHAP values, and the top k features were passed forward, with k treated as a tunable hyperparameter. The final model for each outer fold was refitted on the full outer training data with the hyperparameters and features selected through randomized grid search, then evaluated on the held-out outer test fold, which prevents information leakage (**Figure 1E**).

For feature analysis, we used SHAP values (24, 25). SHAP values decompose individual predictions into feature-specific contributions, enabling both patient-level and global interpretation of feature importance and interactions. Due to the cross-validation scheme, we acquired 10 sets of SHAP values per patient in the training set from the outer loop. We then averaged these SHAP values for further interpretation.

For the validation analysis, hyperparameter selection for the best-performing algorithm was done on the training set using the same grid and randomized search as in the 10×5-fold nested cross-validation, with 2,500 iterations in a 5-fold cross-validation. We did not perform *post hoc* probability calibration, as the primary comparisons between models and clinical risk scores were based on rank-based metrics (AUCs) and risk group stratifications derived from predicted probability percentiles in the training cohort. Moreover, calibration of the final random forest model on the independent test set was already acceptable, with a Brier score of 0.172 **Supplementary Figure S1B**).

Models were built and evaluated using scikit-learn (30) on Python 3.11 (31). SHAP values were computed using the SHAP package (24, 25).

### Statistical analysis and additional information

Additional statistical analyses were performed to assess the independent prognostic contribution of key immune and inflammatory features and to benchmark model performance against established clinical scores.

To assess whether pre-transplant lymphocyte subsets represented independent risk factors for one-year mortality, we performed a multivariable logistic regression analysis. SHAP values from the best-performing machine-learning model were smoothed using locally weighted scatterplot smoothing (LOWESS) and subsequently used to evaluate non-linear associations between each feature and the model-predicted risk. Specifically, for pre-transplant B, CD4^+^ T, CD8^+^ T cells, and CRP, thresholds were defined at points where the smoothed SHAP values crossed zero, indicating a shift in predictive direction. These thresholds were used to construct binary risk indicators. These risk flags, along with the top remaining SHAP-ranked features (excluding the original continuous lymphocyte counts) and additional covariates selected based on clinical relevance and presumed interaction potential, were included in a multivariable logistic regression model fitted on the combined dataset. To evaluate the incremental contribution of risk variables, reduced models excluding these flags were also constructed. Likelihood ratio tests were then performed to compare the full and reduced models.

To benchmark the predictive performance of the random forest model against established clinical risk scores, we compared ROC-derived AUC and test-set risk stratifications. Specifically, the mean AUC from the outer folds of the nested cross-validation (± standard deviation) was used as the unbiased estimate of model performance on the training data, while test-set AUCs were computed on the independent holdout cohort restricted to feature-complete cases. For the clinical scores (HCT-CI, rDRI, EASIX, and mGPS), AUCs were calculated separately on the training and test set. For interpretability, test-set patients were divided into three or four risk groups (tertiles or quartiles) based on predicted probabilities from the training set, and Kaplan-Meier curves were generated to visualize overall survival across risk strata in comparison with the respective clinical scores.

Writing assistance was provided using large language model-based tools to improve clarity and readability under full author supervision.

## Results

### Patient cohort

We included 909 patients who underwent alloHCT between 2008 and 2023, with a median follow-up of survivors of 2,256 days (**Table 1**). The cohort was broadly distributed in age and gender, with acute myeloid leukemia (AML) as the leading diagnosis, followed by myelodysplastic neoplasia (MDS), lymphoma, and multiple myeloma. A majority of patients had active disease at the time of transplant. Matched unrelated and related donors with peripheral blood stem cell grafts were most often used. One third of patients experienced acute graft-versus-host disease (GvHD) beyond grade I, and a similar proportion developed moderate to severe chronic GvHD. During follow-up, about half of the patients died, with disease progression (PD) and infections accounting for most deaths. Of these, 232 patients (26%) died within the first year after alloHCT. Among these early deaths, 102 were due to PD (44%) and 91 (39%) were due to infections.

We tested a total of 31 clinical, immunological, inflammatory, and transplant-related variables for association with one-year mortality after alloHCT (**Table 2**). Of these, a substantial number showed statistically significant differences between survivors and non-survivors, underscoring the complexity of risk prediction. Patients who died within the first year were significantly older and had poorer pre-transplant performance status, elevated LDH, CRP, and creatinine levels, and lower platelet and albumin levels. Regarding immune status, lower pre-transplant counts of B cells, CD4^+^ and CD8^+^ T-cells were significantly associated with one-year mortality. Disease-related factors also contributed: active disease at the time of transplantation was more common among non-survivors, and patients with lymphoma were significantly overrepresented in the early death group. Transplant-related factors also differed between groups: patients who died within one year more often underwent HLA-mismatched transplantation, while GvHD prophylaxis with anti-T lymphocyte globulin (ATLG) was used more frequently in survivors. Use of post-transplant cyclophosphamide (PT-CY) showed a nonsignificant trend toward more frequent application in the early mortality group.

Given the number of significant associations, potential interactions, and non-linear relationships, classical multivariable regression would be limited for this cohort. Therefore, we chose machine learning-based modeling approaches, which are better suited to capture complex, high-dimensional, and non-linear patterns in clinical data.

### Random forest outperforms other machine-learning models in predicting one-year mortality after alloHCT

The goal of our modeling approach was to predict one-year mortality after alloHCT as a binary outcome. Model performance was evaluated using a 10× repeated 5-fold nested cross-validation on the training set (75% of the data, **Figure 1**), with the remaining 25% reserved as an independent test set.

**Figure 2:**
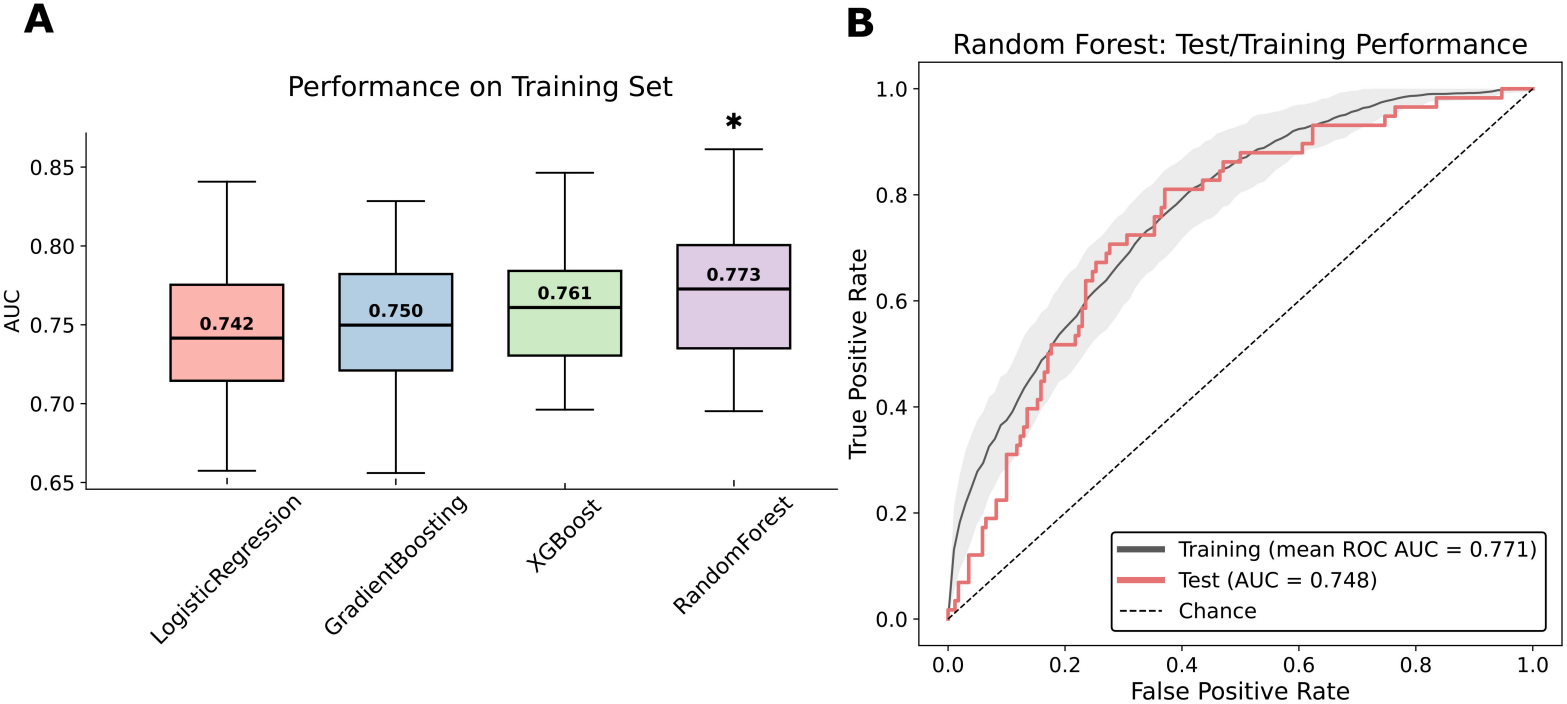
Model performance comparison and validation. **(A)** Area under the curve (AUC) values from 10× repeated 5-fold cross-validation for logistic regression, gradient boosting, XGBoost, and random forest (* p < 0.01 for all comparisons with the random forest). **(B)** Receiver operating characteristic (ROC) curves for the random forest model. The gray line shows the mean ROC across 50 folds (AUC of mean curve = 0.771; shaded area = ± 1 SD). The solid red line represents the ROC on the independent held-out test set (AUC = 0.748).

Across all 50 outer folds, random forest achieved the highest median AUC (0.773), followed by XGBoost (0.761), gradient boosting (0.750), and logistic regression (0.742), as shown in **Figure 2A** (adjusted p < 0.01 for all comparisons with the random forest). A Friedman test showed a significant overall difference (p < 0.01). Pairwise Wilcoxon tests (Bonferroni-corrected) indicated that XGBoost and random forest each outperformed logistic regression (p < 0.05), and random forest also outperformed gradient boosting and XGBoost (p < 0.01). Based on its superior performance, random forest was selected as the final model for our SHAP value analysis.

When applied to the test set, the random forest model maintained robust performance, achieving an AUC of 0.748, indicating good generalizability (**Figure 2B**). Decision curve analysis further confirmed the clinical utility of the model, showing consistent net benefit across relevant threshold probabilities in both the training and test set (**Supplementary Figure S1C**). Calibration of the final random forest model was assessed on the independent test set. The calibration curve demonstrated good alignment in the low-to-intermediate risk range, with a tendency toward modest overestimation at higher predicted probabilities (Brier score of 0.172, calibration intercept −0.14; slope 1.59; **Supplementary Figure S1B**).

To assess whether median imputation influenced model performance, we performed a complete-case sensitivity analysis restricted to patients without missing predictor values (training: 672/681; test: 224/228). Restricting to complete cases resulted in negligible changes in discrimination (ΔAUC +0.0002 in the training set and −0.0018 in the independent test set), indicating that the low level of missing data and the imputation strategy did not affect performance.

### SHAP values illustrate feature importance for outcome prediction

**Figure 3:**
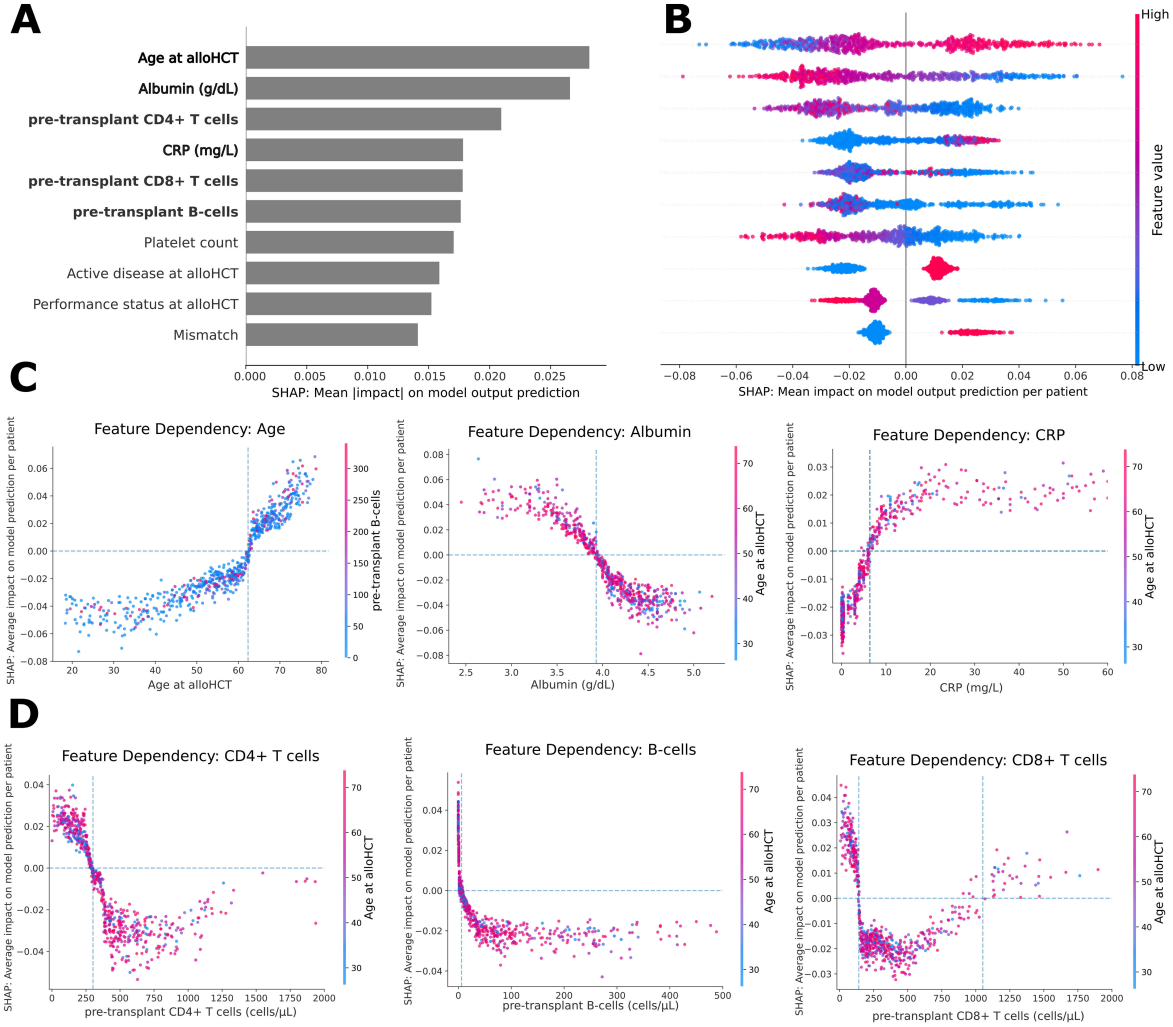
Feature importance and SHAP-based interpretation of the random forest model. **(A)** Bar plot of the mean absolute SHAP values for the top ten features across all ten cross-validation repeats. The SHAP value represents the average magnitude of each feature’s contribution to predicting death within the first year after alloHCT. **(B)** SHAP summary plot showing per-patient SHAP values for each of the top 10 features. Each point represents one patient, with color indicating the feature value (red = high, blue = low). Points to the right of the vertical axis contribute to the prediction of death within the first year, while those on the left contribute against it. **(C, D)** SHAP dependence plots. Each dot represents one patient. SHAP values (y-axis) indicate the direction and magnitude of each feature’s contribution to the model output, while the x-axis shows raw feature value. Dashed vertical and horizontal lines denote observed inflection points, suggesting threshold effects. Color gradients represent interacting feature values. **(C)** Age at alloHCT, pre-transplant albumin, and C-reactive protein (CRP) levels, showing their influence on predicted one-year mortality. **(D)** Pre-transplant lymphocyte subpopulations (CD4^+^, CD8^+^, and B-cells), highlighting non-linear associations with outcome.

SHAP values were used to quantify each feature’s contribution to the predicted probability of death within the first year after alloHCT (**Figure 3**).

The feature with the highest impact was age at alloHCT (mean |SHAP| = 0.028), followed by pre-transplant albumin levels (0.027) and pre-transplant CD4^+^ T-cell count (0.021), CRP levels (0.018), CD8^+^ T-cell count (0.018), and B-cell count (0.018). Notably, three immune cell subsets (CD4^+^, CD8^+^, and B cells) and two inflammatory markers (albumin and CRP) accounted for five of the six most influential predictors, all of which were quantitative and non-disease-specific (**Figure 3A**, **Supplementary Table S2**).

At the individual patient level (**Figure 3B**), higher age, lower albumin, higher CRP, and deviations in pre-transplant CD4^+^, CD8^+^, and B-cell counts contributed most strongly to predicted one-year mortality. SHAP dependence plots revealed distinct patterns: age and albumin showed approximately linear associations with risk, CRP exhibited a plateau effect at higher values, and lymphocyte subsets demonstrated non-linear relationships (**Figures 3C, D**). In particular, low B-cell counts were associated with a steep increase in risk, while CD4^+^ and CD8^+^ T-cell counts displayed U-shaped relationships, with increased risk at both low and high values. These U-shaped relationships persisted across disease categories (**Supplementary Figure S2A**).

These patterns were consistently observed across all tree-based models, and SHAP analysis of the final random forest on the independent test set showed similar feature rankings and dependency curves, supporting the robustness of these findings.

To assess the independent prognostic value of immune and inflammatory features, we fitted a multivariable logistic regression model using one-hot-encoded indicators derived from SHAP-defined thresholds for CD4^+^, CD8^+^, and B-cell counts, as well as CRP (**Supplementary Figure S2B**, **Supplementary Table S2**), capturing the nonlinear relationships observed in the SHAP dependency plots. These indicators remained independently associated with one-year mortality after adjustment for clinically relevant covariates (**Supplementary Figure S2C**). Excluding all three lymphocyte indicators significantly worsened model fit (LR = 24.3, df = 3, p < 0.0001), as did removal of CRP and albumin (LR = 12.1, df = 2, p < 0.01).

### Random forest outperforms established clinical risk scores in prediction and risk discrimination

**Figure 4:**
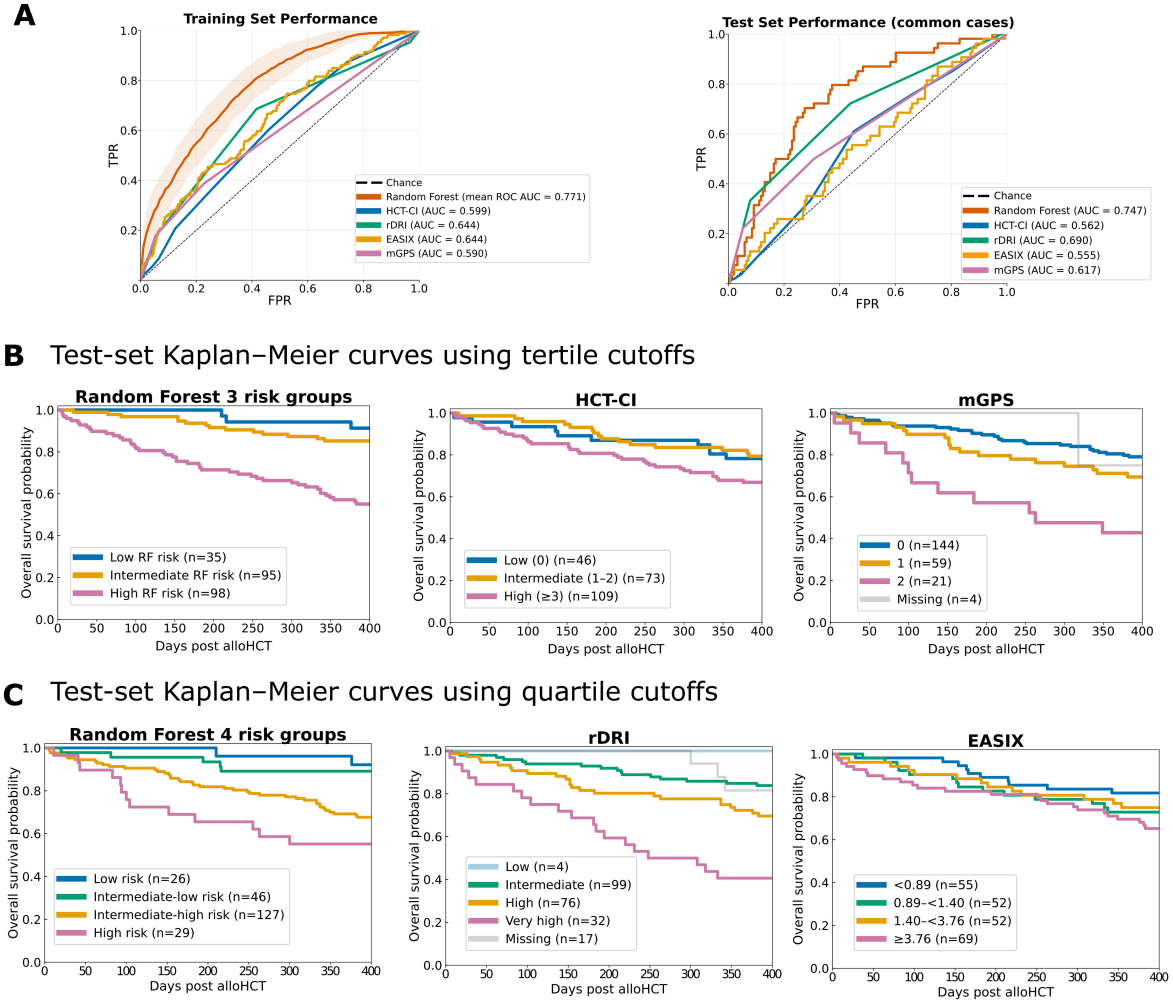
Model performance and model comparison to established clinical scores. **(A)** Receiver operating characteristic (ROC) curves summarizing model discrimination. The left panel shows the mean nested cross-validation ROC for the random forest (RF) on the training set (shaded area = ± 1 SD) compared with clinical scores (HCT-CI, rDRI, EASIX, and mGPS). The right panel shows the ROC curves on the independent test set restricted to feature-complete cases without missing values. **(B)** Kaplan-Meier curves for RF, HCT-CI, and mGPS. For RF, test-set Kaplan-Meier curves were generated using three risk groups (tertiles) defined from training-set predicted probabilities. **(C)** Kaplan-Meier curves for RF, rDRI, and EASIX. For RF, test-set Kaplan-Meier curves were generated using four risk groups (quartiles) defined from the training set.

To benchmark our random forest (RF) model against established clinical risk scores, we compared its performance with HCT-CI, rDRI, EASIX, and mGPS. ROC analysis demonstrated that the RF model clearly outperformed all four scores in both the training and independent test sets (**Figure 4A**). In the nested cross-validation of the training set, the RF achieved the highest mean area under the ROC curve (AUC = 0.771), whereas the clinical scores showed lower discriminatory power (HCT-CI AUC = 0.599, rDRI AUC = 0.644, EASIX AUC = 0.644, and mGPS AUC = 0.590). When applied to the independent test set, the RF model retained robust generalizability with an AUC of 0.747, again exceeding the performance of all established scores. Interestingly, rDRI performed comparatively better on the test set (AUC = 0.690) than in the training set, while HCT-CI (AUC = 0.562), EASIX (AUC = 0.555), and mGPS (AUC = 0.617) remained substantially lower.

Beyond overall discrimination, we evaluated the model’s ability to stratify survival risk within the test cohort (**Figures 4B, C**). To ensure comparability with conventional scoring systems that categorize patients into three risk levels, we defined RF-based tertiles of predicted mortality probability from the training set and compared them directly with HCT-CI and mGPS (**Figure 4B**). The RF model clearly separated test-set patients into three distinct survival trajectories (global log-rank p < 0.001), outperforming both HCT-CI (p = 0.26) and mGPS (p < 0.001). Compared with these scores, the RF identified a true low-risk group with a one-year overall survival of 94% and a clearly defined high-risk group with only 57% survival. In contrast, the corresponding low-risk groups of HCT-CI and mGPS showed substantially lower survival rates of 78% and 81%, respectively. This highlights the RF model’s ability to distinguish both patients with excellent post-transplant outcomes and those at markedly increased risk of early mortality after alloHCT.

When extending the analysis to four groups to mirror the four-tiered risk stratifications of the rDRI and EASIX, the RF model again demonstrated a clear and stepwise separation across all strata (global log-rank p < 0.001; **Figure 4C**, left). One-year overall survival declined progressively from 96% in the lowest-risk quartile (Q1) to 55% in the highest-risk quartile (Q4), illustrating the model’s ability to reliably identify both patients at very low and very high risk of post-transplant mortality. Among the comparator scores, rDRI showed overall significance (p < 0.001; **Figure 4C**, middle) and successfully identified a very-high-risk group with poor outcomes (1-year OS 41%). However, in our test population, rDRI did not define a genuine low-risk group, as only four patients were classified as low risk, all of whom survived beyond one year. In contrast, EASIX produced only modest separation (p = 0.03; **Figure 4C**, right), with overlapping survival between intermediate groups (1-year OS 82-70%).

Taken together, the RF model demonstrated both superior predictive performance and more meaningful clinical stratification than HCT-CI, rDRI, EASIX, or mGPS. Notably, the RF approach is largely based on routine laboratory parameters and remains disease-agnostic, heavily relying on immunological and inflammatory features. By leveraging these predictors, the RF reliably identified patients with excellent one-year survival as well as those with markedly elevated early mortality risk, offering a refined and potentially easily reproducible framework for individualized risk assessment after alloHCT (**Figures 4A–C**). Complementary Kaplan-Meier analyses based on out-of-fold training predictions confirmed consistent risk-group separation (**Supplementary Figure S1D**).

## Discussion

Accurately predicting mortality after alloHCT is a long-standing challenge. Conventional scores offer quick assessments but capture only a small slice of the complex interaction between recipient, disease, and transplant factors, and their performance has not kept pace with changes in conditioning regimens, donor selection, and supportive care. A recent registry-wide machine-learning (ML) effort has shown that flexible algorithms outperform rule-based scores in this setting (12).

By analyzing 31 routinely available pre-transplant variables in 909 recipients with diverse hematologic malignancies, our study demonstrates that machine-learning approaches can improve predictive accuracy. Importantly, these models also identify clinically meaningful and biologically interpretable features. Among the algorithms tested, the random forest model achieved the best discrimination, with a median AUC of 0.773 in cross-validation and 0.748 on the independent test set, outperforming established clinical scores, and demonstrating performance highly competitive with other machine-learning models reported in the alloHCT field (11–13, 32). Given the exceptional biological and clinical complexity of allogeneic transplantation, where outcomes are shaped by intricate interactions between patient, disease, donor, and procedural factors, this improvement in discrimination represents a meaningful advance and highlights the robustness and clinical relevance of the model. SHAP interpretation ranked immune and inflammatory markers such as pre-transplant CD4^+^, CD8^+^, B-cell counts, albumin, and CRP together with patient age at transplantation as the six most influential predictors of one-year mortality. Remarkably, all these parameters are objective, disease-agnostic laboratory measures that are readily available in routine pre-transplant evaluation, underscoring their potential for broad clinical applicability. Our random forest model driven by immunological and inflammatory predictors outperformed established composite indices such as the HCT-CI, rDRI, EASIX, or mGPS. The independent prognostic relevance of lymphocyte subsets and inflammatory markers was further supported by multivariable analysis. This work highlights the exploratory power of machine-learning approaches to uncover clinically relevant, data-driven patterns beyond the scope of conventional risk models (16, 17).

In selecting our modeling framework, we deliberately focused on binary classification algorithms rather than time-to-event models such as random survival forests. Because our follow-up was complete for the first year after transplantation with no censoring before the one-year endpoint, a binary framework was both statistically appropriate and computationally efficient. Moreover, this approach enabled the use of TreeSHAP (25), which allows consistent and interpretable estimation of feature importance across nested cross-validation folds, ensuring generalizable insights into the drivers of risk. Comparable interpretability is currently not feasible for survival-based models or deep-learning approaches within such a rigorous validation design. Thus, tree-based classification models offered an optimal balance between transparency, reproducibility, and computational efficiency without loss of clinically relevant information.

Our work is based on data from a single-center cohort, which may limit generalizability to broader populations. The majority of patients received PBSC grafts and ATLG-based serotherapy, and our cohort tended to be older with more active disease, which may differ from other transplant populations (1, 33). To partly mitigate such center-specific bias, we implemented a rigorous nested cross-validation framework and an independent hold-out test set, ensuring robust internal validation. This strategy increases confidence that the identified predictive patterns reflect true biological and clinical relationships. Hence, our findings support the integration of simple pre-transplant immune and inflammatory profiling into standard alloHCT evaluation to improve risk stratification and guide clinical decision-making, thereby facilitating much-needed multicenter validation and broader implementation.

As the importance of CD4^+^, CD8^+^, and B-cell counts as predictors of mortality after alloHCT is a novel finding, the biological relevance of these pre-transplant immune markers warrants particular attention. Low lymphocyte counts likely reflect cumulative treatment burden, disease activity, and immune senescence. Conversely, high T-cell counts may indicate dysregulated or exhausted immunity. Either extreme could compromise early engraftment, infection control, or graft-versus-leukemia effects. Such non-linear relationships are difficult to capture with classic scores but become visible in SHAP dependence plots and similar tools for model explanation (17). These results suggest that simple flow-cytometry-based immune profiling adds an informative dimension to pre-transplant evaluation that is not reflected in existing risk algorithms. This finding also introduces a new perspective on immune profiling in transplant hematology. Whereas most work to date centers on post-transplant immune reconstitution and its association with survival benchmarks (21, 34–36), our findings emphasize the value of baseline immune competence.

Our machine-learning approach achieved good performance by incorporating the granularity of lymphocyte subsets. In contrast, total lymphocyte counts alone would not capture the complex, non-linear relationships between B- and T-cell levels and survival. Natural killer (NK) cell counts, however, were not identified as important predictors in our models. A possible explanation is that NK cell reconstitution occurs very rapidly after alloHCT (21, 37), with the newly emerging NK cell pool consisting entirely of donor-derived cells, making pre-transplant recipient NK levels potentially less impactful for post-transplant survival.

Beyond immunological factors, our results underscore the growing recognition of systemic inflammation as a key determinant of post-transplant outcome. Markers such as CRP and albumin, established as powerful prognostic indicators in solid malignancies through the modified Glasgow Prognostic Score (mGPS) framework (10), have recently been validated in the alloHCT setting (9). In our work, integrating these inflammatory markers into a machine-learning framework alongside immune cell subsets not only reaffirmed their prognostic relevance but also highlighted their strong, quantifiable impact on early post-transplant mortality. This convergence of evidence from conventional and data-driven approaches emphasizes that systemic inflammation represents a biologically and clinically robust axis of risk-one that complements immunological competence and enhances prediction in alloHCT.

In summary, our disease-agnostic machine-learning model, built from routine baseline data, improves one-year mortality prediction after alloHCT and highlights pre-transplant lymphocyte counts (CD4, CD8, B lymphocytes) and systemic inflammatory markers (CRP, albumin) as complementary risk factors. Identifying and confirming such emerging predictors will sharpen pre-transplant risk assessment and support better shared decision-making between clinicians and patients. In clinical practice, this approach may facilitate more individualized pre-transplant counseling and risk-adapted planning of conditioning intensity, donor selection, and prophylactic or supportive care strategies. Future work will focus on external validation of this model in independent multicenter cohorts to confirm generalizability and to evaluate its feasibility for integration into routine pre-transplant risk assessment. Moreover, these findings will stimulate mechanistic research into how pre-existing immune and inflammatory states shape post-transplant outcomes, potentially uncovering new biological targets for intervention.

## Data Availability

The data analyzed in this study is subject to the following licenses/restrictions: The dataset contains sensitive personal health information from our institutional clinical database and cannot be fully anonymized under local data protection regulations. Access can therefore only be granted upon reasonable request. Requests to access these datasets should be directed to claudia.wehr@uniklinik-freiburg.de.
