# Supplementary material for: Machine learning-based prediction of one-year mortality after alloHCT identifies the impact of pre-transplant immunity and inflammation
Source: Front Immunol. 2026 Jan 19;16:1745873. doi: 10.3389/fimmu.2025.1745873 (PMC12861908; doi:10.3389/fimmu.2025.1745873)
Supplement: Supplementary file 1 [file DataSheet1.docx]

Supplementary Figure Legends

Supplemental Figure 1. Model calibration, decision analysis, and training-set Kaplan-Meier curves.

(A) Flow diagram of cohort assembly. Of 1,346 first alloHCT recipients (2008–2023), 437 were excluded for missing lymphocyte subset data, leaving 909 patients split into training (75%, n = 677) and test (25%, n = 232) sets.
(B) Calibration curve for one-year mortality showing observed versus predicted event rates across ten probability bins. Good overall calibration with a Brier score of 0.172.
(C) Decision curve analysis (DCA) for the RF model on the training (Train, left panel) and independent test sets (Test, right panel). Net benefit is plotted across clinically relevant threshold probabilities (0.1-0.4). For the training set, out-of-fold predicted probabilities were averaged across nested cross-validation repeats to obtain unbiased estimates.
(D) Kaplan-Meier curves on the training set comparing survival stratification by RF-derived risk groups (tertiles and quartiles) with established clinical scores (HCT-CI, mGPS, rDRI, and EASIX)

Supplemental Figure 2. Threshold definition and multivariable analysis.

(A) SHAP dependence plots for pre-transplant CD8⁺ T-cell (left) and CD4⁺ T-cell counts (right), with individual patients colored by underlying disease category. In addition to the disease categories explicitly modeled in the multivariable analysis (AML, MDS, lymphoma), the visualization also includes additional diagnoses (multiple myeloma, MPN, ALL, and other) to provide a comprehensive view of disease distribution. The persistence of U-shaped, non-linear associations across diagnoses indicates that the elevated risk observed at higher lymphocyte counts is not driven by a single disease entity.

(B) SHAP-based partial dependence plots for pre-transplant CD4⁺ T cells, CD8⁺ T cells, B cells, and CRP, derived from the random forest model in the training set. Each dot represents an individual patient, with SHAP values on the y-axis indicating the feature’s contribution to predicted one-year mortality. LOWESS smoothing curves were used to define thresholds at inflection points where the direction of predictive impact changed.

(C) Multivariable logistic regression model including binary risk indicators derived from thresholds in (B) together with the top SHAP-ranked variables and clinically relevant disease categories (AML, MDS, lymphoma). The forest plot displays odds ratios (ORs) with 95% confidence intervals (CIs)
