# Supplementary figures and images for: Machine learning-based prediction of one-year mortality after alloHCT identifies the impact of pre-transplant immunity and inflammation
Source: Front Immunol. 2026 Jan 19;16:1745873. doi: 10.3389/fimmu.2025.1745873 (PMC12861908; doi:10.3389/fimmu.2025.1745873)

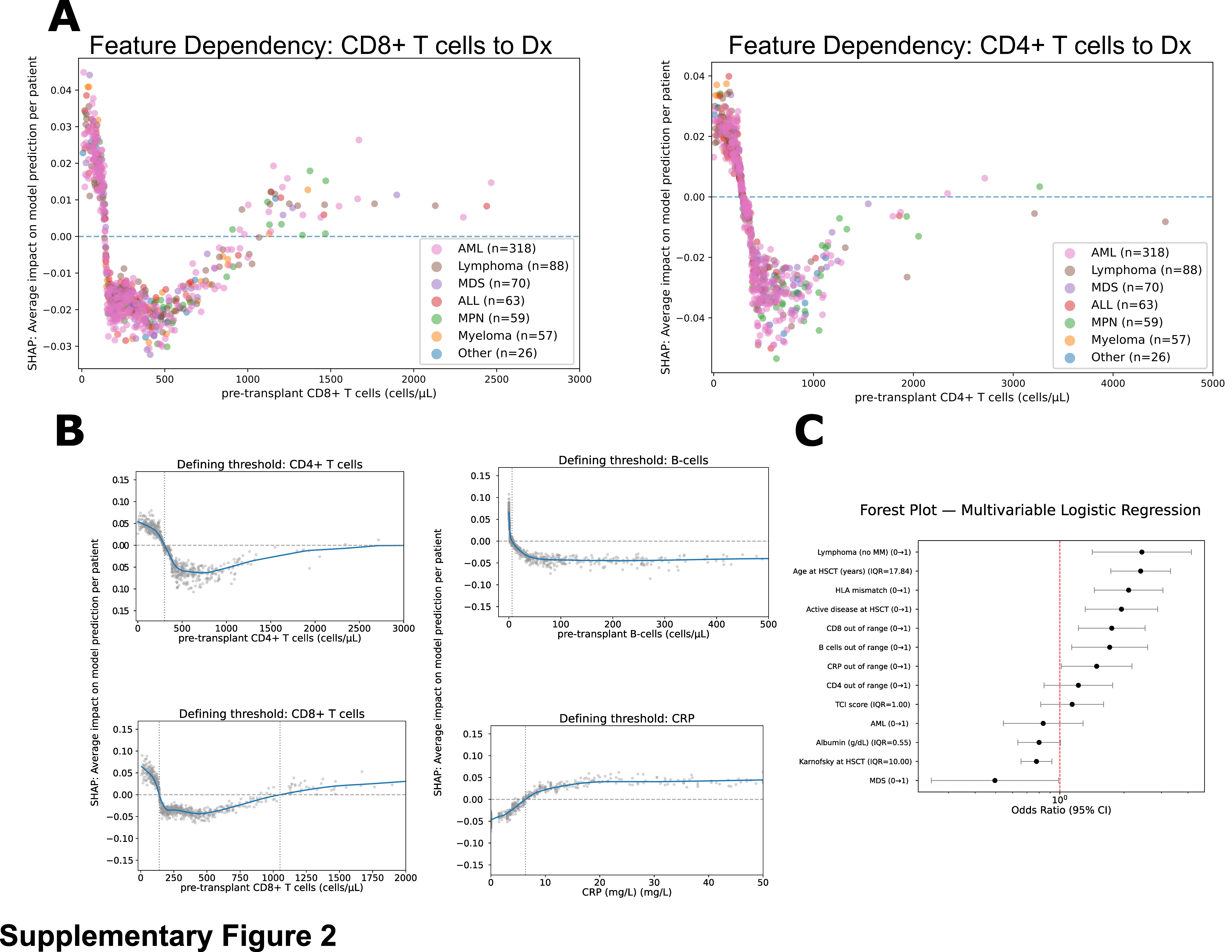

Supplement: Supplementary file 3 [file Image2.jpeg]
