# Supplementary material for: Machine learning-based prediction of one-year mortality after alloHCT identifies the impact of pre-transplant immunity and inflammation
Source: Front Immunol. 2026 Jan 19;16:1745873. doi: 10.3389/fimmu.2025.1745873 (PMC12861908; doi:10.3389/fimmu.2025.1745873)
Supplement: Supplementary file 4 [file Table1.docx]

| **Section** | **Checklist item** | | | **Reported in section** | | | **Short summary** | |
| --- | --- | --- | --- | --- | --- | --- | --- | --- |
| Title | Identify the study as developing or evaluating the performance of a multivariable prediction model, the target population, and the outcome to be predicted | | | Title | | | Title identifies the study as a machine-learning-based development of a multivariable prediction model for one-year mortality in alloHCT recipients. | |
| Background | Explain the healthcare context (including whether diagnostic or prognostic) and rationale for developing or evaluating the prediction model, including references to existing models | | | Introduction | | | Introduction outlines the prognostic context of alloHCT with persistent one-year mortality and limited accuracy of existing prognostic scores. Previous studies using conventional and ML approaches are summarized to show gaps in addressing pre-transplant immune and inflammatory status. The study aims to develop an explainable ML model integrating immunological and inflammatory predictors to improve individualized risk estimation. | |
|  | Describe the target population and the intended purpose of the prediction model in the context of the care pathway, including its intended users (e.g., healthcare professionals, patients, public) | | | Methods | | | The target population comprises adult patients undergoing first alloHCT for hematologic malignancies. The model aims to predict one-year mortality risk and identify novel pre-transplant risk factors, particularly immunological and inflammatory markers. Intended audience includes researchers and clinicians interested in uncovering new prognostic factors. | |
|  | Describe any known health inequalities between sociodemographic groups | | |  | | | This was a single-center study conducted at a German university transplant center. Therefore, findings reflect the local healthcare setting, where access to alloHCT follows standardized national criteria. Broader sociodemographic health inequalities were not specifically analyzed, but general limitations regarding representativeness of this population apply | |
| Objectives | Specify the study objectives, including whether the study describes the development or validation of a prediction model (or both) | | | Methods | | | Study objective: Develop a machine-learning-based multivariable prediction model for one-year mortality after alloHCT. | |
| Data | Describe the sources of data separately for the development and evaluation datasets (e.g., randomized trial, cohort, routine care or registry data), the rationale for using these data, and representativeness of the data | | | Methods | | | Data source: Single-center retrospective cohort from a prospectively maintained institutional alloHCT database (University Medical Center Freiburg, 2008-2023).  Dataset split: 75/25 stratified train-test division for model development and internal evaluation, maintaining event balance and avoiding data leakage.  Rationale: Comprehensive, high-quality dataset with uniform diagnostic, laboratory, and transplant procedures, enabling consistent measurement of immunological and clinical predictors.  Representativeness: Reflects a transplant population; findings may require validation in more diverse, multicenter cohorts | |
|  | Specify the dates of the collected participant data, including start and end of participant accrual; and, if applicable, end of follow-up | | | Methods | | | See point above | |
| *Participants* | Specify key elements of the study setting (e.g., primary care, secondary care, general population)  including the number and location of centres | | | Methods | | | See point above | |
|  | Describe the eligibility criteria for study participants | | | Methods | | | Included all adult patients undergoing first alloHCT between 2008 and 2023 at our center with available pre-transplant lymphocyte subset data in the institutional database. | |
|  | Give details of any treatments received, and how they were handled during model development or evaluation, if relevant | | | Methods | | | All patients received first alloHCT for hematologic malignancies.  Treatment variables were used as recorded to reflect real-world practice. | |
| *Data preparation* | Describe any data pre-processing and quality checking, including whether this was similar across relevant sociodemographic groups | | | Methods | | | Standardized institutional data with uniform preprocessing (one-hot encoding, median imputation).  Pre-transplant labs taken day -20 to -6 for all patients.  Same procedures applied across all demographic groups. | |
| *Outcome* | Clearly define the outcome that is being predicted and the time horizon, including how and when assessed, the rationale for choosing this outcome, and whether the method of outcome assessment is consistent across sociodemographic groups | | | Methods | | | Outcome: Death within the first year after alloHCT (binary endpoint).  Assessment: Determined from institutional clinical records with complete follow-up.  Rationale: One-year mortality is a clinically meaningful endpoint with sufficient events and a fixed, binary outcome- making it well-suited for tree-based models with SHAP interpretation.  Consistency: Outcome assessment uniform across all patients and demographic groups. | |
|  | | | If outcome assessment requires subjective interpretation, describe the qualifications and demographic characteristics of the outcome assessors | |  | Not applicable | | |
|  | | | Report any actions to blind assessment of the outcome to be predicted | |  | Not applicable: Outcome data (one-year mortality) were obtained retrospectively from institutional records after all treatments had occurred; therefore, blinding was not required or applicable. | | |
| *Predictors* | | | Describe the choice of initial predictors (e.g., literature, previous models, all available predictors) and any pre-selection of predictors before model building | | Methods | Predictor selection: Based on prior literature, established transplant risk scores, clinical relevance and pre-transplant immunological and inflammatory markers  Included 31 routinely available pre-transplant variables  No automated pre-selection performed; all features entered the model pipeline, | | |
|  | | | Clearly define all predictors, including how and when they were measured (and any actions to blind assessment of predictors for the outcome and other predictors) | | Methods | Predictors: 31 pre-transplant variables covering patient, donor, disease, immune, and laboratory factors.  Measurement timing: All laboratory and lymphocyte subset data obtained uniformly between day -20 and -6 before conditioning.  Assessment: Data drawn from standardized institutional records and flow cytometry measurements. | | |
|  | | | If predictor measurement requires subjective interpretation, describe the qualifications and demographic characteristics of the predictor assessors | | Methods | Subjective predictors such as Karnofsky score assessed by transplant physicians using standardized institutional and published criteria | | |
| *Sample size* | | | Explain how the study size was arrived at (separately for development and evaluation), and justify that the study size was sufficient to answer the research question. Include details of any sample size calculation | | Methods | Design & size: Retrospective complete-case cohort; all eligible first alloHCTs with required data (n=909). | | |
| *Missing data* | | | Describe how missing data were handled. Provide reasons for omitting any data | | Methods | Missing data were minimal (<0.001%) and handled by median imputation for all models except XGBoost, which natively manages missing values; no data were omitted | | |
| *Analytical methods* | | | Describe how the data were used (e.g., for development and evaluation of model performance) in the analysis, including whether the data were partitioned, considering any sample size requirements | | Methods/Figure 1 | Data split 75/25 (train/test) using stratified sampling to preserve event distribution (~25% mortality).  Training set: Used for model development, feature selection, and hyperparameter tuning via 10×5-fold nested cross-validation.  Test set: Held out entirely for final model evaluation to ensure unbiased performance assessment. | | |
|  | | | Depending on the type of model, describe how predictors were handled in the analyses (functional form, rescaling, transformation, or any standardisation). | | Methods | Encoding: Categorical predictors one-hot encoded; continuous predictors kept on their native scale (no standardization).  Missingness: Median imputation inside the pipeline (except XGBoost, which natively handles missing values).  Functional form: No explicit transformations; tree models capture non-linearities natively. Logistic regression models a linear log-odds relation on the selected features (not explicitly mentioned in Methods).  Logistic regression details (not explicitly mentioned in Methods).: Explored L1/L2/elastic-net penalties (liblinear/saga), C via randomized search; optional class_weight='balanced';. | | |
|  | | | Specify the type of model, rationale, all model-building steps, including any hyperparameter tuning, and method for internal validation | | Methods | Model types: Logistic regression, random forest, gradient boosting, and XGBoost.  Rationale: Compared linear and tree-based algorithms to balance interpretability and predictive performance for one-year mortality.  Model-building steps: Implemented a nested cross-validation pipeline, randomized hyperparameter search, and median imputation within each fold to prevent data leakage.  Hyperparameter tuning: Performed using randomized search with ROC AUC scoring.  Internal validation: Conducted via 10×5-fold nested cross-validation and an independent 25% hold-out test set for unbiased performance estimation. | | |
|  | | | Describe if and how any heterogeneity in estimates of model parameter values and model performance was handled and quantified across clusters (e.g., hospitals, countries). See TRIPOD-Cluster for additional considerations | | Methods | Not applicable: Single-center study; no clustering by hospital or country. Therefore, heterogeneity of model parameters or performance across clusters was not assessed. | | |
|  | | | Specify all measures and plots used (and their rationale) to evaluate model performance (e.g., discrimination, calibration, clinical utility) and, if relevant, to compare multiple models | | Methods, Figure 2 & 3 | Model performance: Evaluated primarily by AUC for discrimination.  Calibration: Not required for rank-based model comparison; overall Brier score for the final model was 0.172, indicating good probabilistic accuracy.  Model comparison: AUCs compared across algorithms using cross-validation with Bonferroni-adjusted pairwise tests.  Visualization: ROC and Kaplan-Meier curves illustrated discrimination and clinical risk stratification. | | |
|  | | | Describe any model updating (e.g., recalibration) arising from the model evaluation, either overall or for particular sociodemographic groups or settings | | Methods | Not applicable: No model updating or recalibration was performed; results reflect internally validated models from the original development cohort. | | |
|  | | | For model evaluation, describe how the model predictions were calculated (e.g., formula, code, object, application programming interface) | | Methods | Model predictions were computed using the trained ML models in scikit-learn (Python 3.11) within the nested cross-validation pipeline | | |
| *Class imbalance* | | | If class imbalance methods were used, state why and how this was done, and any subsequent methods to recalibrate the model or the model predictions | | Methods | The dataset showed moderate class imbalance (~25% one-year mortality).  To address this, class_weight='balanced' was included as a tunable hyperparameter in the logistic regression and tree-based models.  No further recalibration of predicted probabilities was required, as discrimination was the primary evaluation metric. | | |
| *Fairness* | | | Describe any approaches that were used to address model fairness and their rationale | |  | Not applicable: The study focused on clinical and biological predictors within a single-center cohort; no explicit fairness assessment or mitigation was required, as sociodemographic bias was not part of the study question | | |
| *Model output* | | | Specify the output of the prediction model (e.g., probabilities, classification). Provide details and rationale for any classification and how the thresholds were identified | | Methods | Model outputs: Predicted probabilities of one-year mortality and corresponding SHAP values quantifying each feature’s contribution to individual predictions.  Classification: Patients stratified into risk groups (tertiles and quartiles) based on predicted probabilities from the training cohort.  Rationale: Probabilities enable individualized risk estimation, while SHAP values provide transparent feature-level interpretation; quantile grouping supports visual and survival-based comparison. | | |
| *Training versus*  *evaluation* | | | Identify any differences between the development and evaluation data in healthcare setting, eligibility criteria, outcome, and predictors | |  | Not applicable: Development and evaluation data originated from the same single-center cohort, using identical eligibility criteria, predictors, and outcome definitions. | | |
| *Ethical approval* | | | Name the institutional research board or ethics committee that approved the study and describe the participant-informed consent or the ethics committee waiver of informed consent | | Methods | Ethics approval: Granted by the Ethics Committee of the University of Freiburg (EK-FR: 22-1490-S1-retro).  Informed consent: Obtained from all patients for data analysis in accordance with the Declaration of Helsinki. | | |
| *Funding* | | | Give the source of funding and the role of the funders for the present study | | Title Page | Funding: The study received no external funding.  Role of funders: Not applicable; the research was conducted independently by the investigators without sponsor involvement in study design, analysis, or manuscript preparation | | |
| *Conflicts of*  *interest* | | | Declare any conflicts of interest and financial disclosures for all authors | | Title Page | See COI section of the MS | | |
| *Protocol* | | | Indicate where the study protocol can be accessed or state that a protocol was not prepared | |  | Protocol: A study protocol was not prepared, as this is a retrospective analysis using data from a prospectively maintained institutional database. | | |
| *Registration* | | | Provide registration information for the study, including register name and registration number, or state that the study was not registered | |  | Not applicable, see above | | |
| *Data sharing* | Provide details of the availability of the study data | | |  | | | Data availability: The data underlying this study are available from the corresponding author, Claudia Wehr, upon reasonable request | |
| *Code sharing* | Provide details of the availability of the analytical code4 | | |  | | | Code availability: The code underlying this study is available from the corresponding author, Claudia Wehr, upon reasonable request | |
| *Patient & Public*  *Involvement* | Provide details of any patient and public involvement during the design, conduct, reporting, interpretation, or dissemination of the study or state no involvement. | | |  | | | Not applicable: Patients or the public were not involved in the design, conduct, analysis, or reporting of this retrospective study. | |
| *Participants* | Describe the flow of participants through the study, including the number of participants with and without the outcome and, if applicable, a summary of the follow-up time. A diagram may be helpful. | | | Results | | | Study population: 909 patients undergoing first alloHCT (2008-2023), selected from 1346 patients undergoing alloHCT in that time frame.  Inclusion criterion: Availability of pre-transplant lymphocyte subset data  Primary outcome: Death within one year after alloHCT  Outcome distribution: 232 deaths (26%), 677 survivors (74%)  Median follow-up: 1,040 days | |
|  | Report the characteristics overall and, where applicable, for each data source or setting, including the key dates, key predictors (including demographics), treatments received, sample size, number of outcome events, follow-up time, and amount of missing data. A table may be helpful. Report any  differences across key demographic groups. | | | Methods, Results, Table 1 and Table 2 | | | Setting: Single-center retrospective study at University Hospital Freiburg  Study period: 2008-2023  Sample size: 909 patients undergoing first alloHCT  Outcome events: 232 deaths (26%) within one year after alloHCT  Key predictors: 31 pre-transplant variables including  Demographics: age, sex,  Laboratory markers: LDH, CRP, creatinine, albumin, ALT, platelets  Immunological parameters: pre-transplant CD4⁺, CD8⁺, B-cell counts and NK cell counts  Disease and transplant characteristics  Treatments received: Standard-of-care alloHCT  Outcome definition: Death within one year after alloHCT  Missing data: Very low overall (0.0005% of all data points);  Key demographic differences: Patients who died within one year were older and had higher CRP and LDH, lower albumin and platelet counts, and lower lymphocyte subset levels compared with survivors | |
|  | For model evaluation, show a comparison with the development data of the distribution of important predictors (demographics, predictors, and outcome). | | | Figure 1, Methods, Results, Table 2 | | | Data split: Stratified 75/25 division into training (n = 681) and test (n = 228) sets  Outcome distribution:  Training set - 172 deaths (25%) and 509 survivors  Test set - 60 deaths (26%) and 168 survivors  Purpose: Stratification ensured balanced outcome frequencies across both sets | |
| *Model development* | Specify the number of participants and outcome events in each analysis (e.g., for model development, hyperparameter tuning, model evaluation | | | Figure 1, Methods, Results | | | Model development: 681 patients (training set)  Outcome events: 172 deaths (25%) within one year  Hyperparameter tuning: Conducted within fold nested cross-validation  Outcome events per outer fold ≈ 25% mortality  Model evaluation (independent test set): 228 patients  Outcome events: 60 deaths (26%) within one year  Total cohort: 909 patients  Overall outcome events: 232 deaths (26%) | |
| *Model*  *specification* | Provide details of the full prediction model (e.g., formula, code, object, application programming interface) to allow predictions in new individuals and to enable third-party evaluation and implementation, including any restrictions to access or re-use (e.g., freely available, proprietary)5 | | | Figure1, Methods, Results | | | Model type: Random forest classifier (Python 3.11, scikit-learn)  Input: 31 pre-transplant clinical, immunological, and inflammatory variables  Output: Predicted one-year mortality probability after alloHCT  Access: Model code and pipeline available on reasonable request due to sensitive nature of the data.  Software: Open-source packages (scikit-learn, SHAP) | |
| *Model*  *performance* | Report model performance estimates with confidence intervals, including for any key subgroups (e.g., sociodemographic). Consider plots to aid presentation. | | | Results | | | Overall performance:  Training (nested CV): AUC = 0.773 ± 0.042  Independent test set: AUC = 0.748  Comparator scores (test AUCs): HCT-CI = 0.562, rDRI = 0.606, EASIX = 0.555, mGPS = 0.617  Risk stratification:  RF tertiles: clear separation (global log-rank p < 0.001)  RF quartiles: stepwise decline in survival (Q1 96% , Q4 55%)  Visualization: ROC curves and Kaplan-Meier plots shown in Figure 4A-C | |
|  | If examined, report results of any heterogeneity in model performance across clusters. See TRIPOD Cluster for additional details3. | | |  | | | Not applicable - the analysis was performed on a single-center cohort without predefined clusters or multiple data sources | |
| *Model updating* | Report the results from any model updating, including the updated model and subsequent performance | | |  | | | Model updating: The current model represents an updated version of a previously presented model (ASH oral presentation, n = 491,  https://doi.org/10.1182/blood-2024-199021 ).  Changes: Expanded dataset (n = 909) and inclusion of additional laboratory parameters (e.g., albumin, CRP).  Performance: Similar predictive accuracy maintained (AUC = 0.773 in training; 0.748 in test) vs.  Key finding retained: Pre-transplant lymphocyte subsets remained among the strongest predictors of one-year mortality. | |
| *Interpretation* | Give an overall interpretation of the main results, including issues of fairness in the context of the objectives and previous studies | | | Discussion | | | Main finding: RF model outperformed clinical scores in predicting one-year mortality after alloHCT.  Key drivers: Pre-transplant immunocompetence and inflammation identified as dominant predictors.  Context: Extends prior ML studies by revealing specific biological determinants outperforming scoring systems | |
| *Limitations* | Discuss any limitations of the study (such as a non-representative sample, sample size, overfitting, missing data) and their effects on any biases, statistical uncertainty, and generalizability | | | Discussion | | | Single-center design: May limit generalizability to other institutions or populations.  Sample size: Moderate (n = 909); external validation needed for broader applicability.  Potential bias: Center-specific practices could influence model feature importance.  Overfitting risk: Minimized through nested cross-validation and independent test set.  Missing data: Minimal (<0.001%) and handled by median imputation; unlikely to affect results. | |
| *Usability of the*  *model in the*  *context of current*  *care* | | Describe how poor quality or unavailable input data (e.g., predictor values) should be assessed and handled when implementing the prediction model | | | Discussion | | | Not applicable - the model is not publicly available for external implementation. |
|  | Specify whether users will be required to interact in the handling of the input data or use of the model, and what level of expertise is required of users | | | Discussion | | | Not applicable - the model is not currently available for public or clinical use.  Note: During development, all input handling was automated within the analysis pipeline. External users would not be required to interact with data preprocessing | |
|  | Discuss any next steps for future research, with a specific view to applicability and generalizability of the model | | | Discussion | | | Next steps: External validation in independent, multicenter cohorts to confirm generalizability.  Model refinement: Incorporate additional biological and clinical variables to enhance predictive scope.  Clinical integration: Prospective evaluation for real-time pre-transplant risk assessment.  Mechanistic exploration: Elucidate immunological and inflammatory pathways underlying the identified risk patterns. | |
